# Melatonin Orchestrates Lipid Homeostasis through the Hepatointestinal Circadian Clock and Microbiota during Constant Light Exposure

**DOI:** 10.3390/cells9020489

**Published:** 2020-02-20

**Authors:** Fan Hong, Shijia Pan, Pengfei Xu, Tingting Xue, Jialin Wang, Yuan Guo, Li Jia, Xiaoxiao Qiao, Letong Li, Yonggong Zhai

**Affiliations:** 1 Beijing Key Laboratory of Gene Resource and Molecular Development, College of Life Sciences, Beijing Normal University, Beijing 100875, China; hongfanky@126.com (F.H.); 15129062355@163.com (S.P.); PEX9@pitt.edu (P.X.); xtt1206@163.com (T.X.); wangjialin9203@163.com (J.W.); gy5326@126.com (Y.G.); 15130160219@163.com (L.J.); 201921200023@mail.bnu.edu.cn (X.Q.); 201921200016@mail.bnu.edu.cn (L.L.); 2 Key Laboratory for Cell Proliferation and Regulation Biology of State Education Ministry, College of Life Sciences, Beijing Normal University, Beijing 100875, China; 3 Center for Pharmacogenetics and Department of Pharmaceutical Sciences, University of Pittsburgh, Pittsburgh, PA 15213, USA

**Keywords:** melatonin, LAN, hepatointestinal, microbiota, lipid homeostasis

## Abstract

Misalignment between natural light rhythm and modern life activities induces disruption of the circadian rhythm. It is mainly evident that light at night (LAN) interferes with the human endocrine system and contributes to the increasing rates of obesity and lipid metabolic disease. Maintaining hepatointestinal circadian homeostasis is vital for improving lipid homeostasis. Melatonin is a chronobiotic substance that plays a main role in stabilizing bodily rhythm and has shown beneficial effects in protecting against obesity. Based on the dual effect of circadian rhythm regulation and antiobesity, we tested the effect of melatonin in mice under constant light exposure. Exposure to 24-h constant light (LL) increased weight and insulin resistance compared with those of the control group (12-h light–12-h dark cycle, LD), and simultaneous supplementation in the melatonin group (LLM) ameliorated this phenotype. Constant light exposure disturbed the expression pattern of a series of transcripts, including lipid metabolism, circadian regulation and nuclear receptors in the liver. Melatonin also showed beneficial effects in improving lipid metabolism and circadian rhythm homeostasis. Furthermore, the LL group had increased absorption and digestion of lipids in the intestine as evidenced by the elevated influx of lipids in the duodenum and decrease in the efflux of lipids in the jejunum. More interestingly, melatonin ameliorated the gut microbiota dysbiosis and improved lipid efflux from the intestine. Thus, these findings offer a novel clue regarding the obesity-promoting effect attributed to LAN and suggest a possibility for obesity therapy by melatonin in which melatonin could ameliorate rhythm disorder and intestinal dysbiosis.

## 1. Introduction

As a disorder of lipid homeostasis, obesity has developed into a global concern; meanwhile, obesity induced by environmental factors has raised attention. A number of studies now focus on the disruption of the endocrine system by chemicals, which subsequently affect weight and tissue function homeostasis [1,2]. In recent decades, increasing research has begun to investigate the effect of light pollution on health [3,4]. Misalignment between physiological processes, which is an outcome of evolution for more than three billion years, and modern life’s artificial night light utilization have become one of the vital reasons for circadian rhythm disorder [5,6]. Shift work and night work are widespread in most industrialized countries and are estimated to be 75% of the total workforce. Moreover, growing exposure to light from intelligent device screens, family and night scenes of the city also aggravate the hazard from artificial night light [4]. A recent study combining satellite images from the US Defense Meteorological Satellite Program with obesity prevalence rates in more than 80 countries from the World Health Organization (WHO) further identified light at night (LAN) as one of the factors contributing to the increasing rates of obesity and metabolic diseases [1,7,8].

The body clock system is regulated by an endogenous oscillator, which is located in the suprachiasmatic nuclei (SCN) of the hypothalamus and responsible for the synchronization of peripheral tissues [3,5]. Light is the most significant exogenous signal for rhythm formation, which involves stimulating light-sensitive ganglion cells in the retina and transferring photic information to the SCN to synchronously produce cyclic electrical activity [9]. Thus, we possess day-night cycle behavior and body physiology [10,11]. A series of proteins participate in body rhythm regulation, including *Bmal1*, *Clock*, *Cry*, *Per*, and other nuclear receptors, which constitute a transcriptional feedback loop [12,13,14]. This cell-autonomous mechanism causes the rhythmicity of metabolism regulation in many physiological processes, including food intake, hormone secretion, and energy metabolism [15]. Misalignment between natural light rhythm and modern life activities, including shift work, travel across time zones and exposure to light at night, induces disruption of the circadian rhythm. In particular, increasing studies have revealed that long-term disruption of circadian rhythm will incur glucose and lipid metabolism disorder and imbalance of energy metabolism [16].

In fact, peripheral tissues such as the liver, gastrointestinal (GI) tract, heart, and other tissues also show circadian oscillations that are coordinated by the SCN [17,18,19]. A growing body of studies has investigated circadian rhythm disruption in the hepatointestinal system associated with various metabolic disorders [20,21,22,23]. The clock transcriptional feedback loop regulates nearly 30% of genes expressed in the hepatointestinal system [21]. The hepatointestinal system mediates nutrient digestion and absorption, which are also influenced by exogenous signals (e.g., food acquisition and light exposure) [20,24]. In patients with nonalcoholic fatty liver disease (NAFLD), single nucleotide polymorphisms in the circadian gene *CLOCK* (rs1801260) and *PPARγ* (rs3856806) are highly prevalent [25,26]. Chronic jet lag has been reported to aggravate steatohepatitis and even induce hepatocellular carcinoma in *Fxr*^-/-^ mice [27]. Regulation of circadian rhythm by posttranslational mechanisms is generally associated with gut permeability, the interconnection between the host and microbiota, immune homeostasis and cellular respiration in the GI tract [22]. Furthermore, the gut microbiome, which serves as a signaling hub between host health and environmental factors such as light rhythm conditions, also plays an important role in the hepatointestinal system [23,28,29]. Recently, Gao T et al. reported that sleep deprivation induced microbiota dysbiosis and intestinal barrier dysfunction [30]. The composition and function of the microbiome are influenced by its environment, and dynamic changes and consequences potentially regulate host body homeostasis.

Melatonin, N-acetyl-5-methoxytryptamine, is an endogenous hormone secreted by the pineal gland, and the plasma level increases during the evening, peaks at night, and decreases to zero during the daytime [31]. But the sensitivity of response to light is characteristic of melatonin. It was suppressed by 50% in plasma when evening light just at <30 lux [32]. Melatonin plays a role in various physiological activities, serving as a gatekeeper of circadian clocks, modulating memory formation and even passing circadian timing cues from mother to infant [30,33,34]. Melatonin improves new bone formation through mediating Wnt4 signaling and increases the uptake of oxidized vitamin C via upregulating Glut1 [35,36]. In addition, melatonin protected against obesity and relative hepatic steatosis by improving lipid dysmetabolism, stimulating brown adipose tissue (BAT) browning, inhibiting epididymal adipocyte-derived exosomal resistin and attenuating inflammation in high-fat diet (HFD)-fed mice [37,38,39,40,41]. Melatonin not only exhibited marked effects on the liver to prevent nonalcoholic steatohepatitis (NASH) and even hepatocellular carcinoma but also showed significant protection in gastrointestinal mucosa [21,42]. Melatonin shows a beneficial effect on the balance between the internal biological clock and food intake [21]. Melatonin receptors found in mouse hepatocytes were one of the reasons for its regulation mechanism [43]. Melatonin receptors are also widely distributed in intestinal tissue, and melatonin can be adsorbed in the duodenum, which indicates that melatonin may regulate this tissue [44]. Recently, increasing studies have demonstrated that supplementing melatonin has the potential to improve the balance between host and commensal bacteria, such as enhancing intestinal barrier stability in sleep deprivation conditions and alleviating weanling stress [30,45]. Paulose JK reported that the GI tract also possesses the ability to secrete melatonin, which inversely regulates the swarming and motility of the microbiome [46]. Furthermore, our previous study demonstrated that melatonin also displayed probiotic ability through reversing HFD-induced gut microbiota dysbiosis, which improved the ratio of *Firmicutes* to *Bacteroidetes* and the richness and abundance of *Akkermansia* [38].

Melatonin is a chronobiotic substance that plays a main role in stabilizing bodily rhythm. Our previous study showed that melatonin prevents body weight gain, alleviates liver steatosis, and reduces low-grade inflammation, as well as improves insulin resistance in HFD-fed mice [38]. However, the effect of it in the hepatointestinal system is still unclear, especially in circadian rhythm regulation of the hepatointestinal system. Based on the dual effect of circadian rhythm regulation and antiobesity, we tested the effect of melatonin in the hepatointestinal system of mice under constant light exposure.

## 2. Materials and Methods

### 2.1. Drug and Diets

Melatonin (M5250), insulin, oleic acid (OA), and palmitic acid (PA) were purchased from Sigma-Aldrich (St. Louis, MO, USA). Bodipy was purchased from Thermo Fisher Scientific (Waltham, MA USA). The HFD (containing 60% kcal from fat) was purchased from Beijing HFK Bioscience Co. Ltd., (Beijing, China), and the detailed composition of diet is shown in the Supplementary Table S1.

### 2.2. Animals and Experimental Design

We bought nine-week-old male C57BL/6J mice from Vital River Laboratory Animal Technology Co. Ltd. (Beijing, China). The mice were randomly assigned to three groups (n = 20–24) in a climate-controlled room at 25 ± 2 °C, with free access to water and high-fat diets after a 1-week adaptation period. The LD group (12h light-12 h dark cycle) was subjected to a 12-h light–12-h dark cycle (lights on at 8:00 a.m. and off at 20:00 p.m.), while the LL group (24 h light) and LLM group (24 h light and treated with melatonin) were exposed to 24-h light conditions. The LLM group was treated with melatonin concentrations at 0.4 mg/mL (50 mg/kg body weight (BW)) in water only began at zeitgeber time (ZT) 12 to ZT24 (treatment began from 20:00 p.m. and stop at 08:00 a.m.) and changed to normal water during ZT0 to ZT12 (from 08:00 a.m. to 20:00 p.m.) daily. After 10 weeks raised or treatment, tissues were collected at ZT2, ZT8, ZT14, and ZT20 (They correspond to 10:00 a.m., 16:00 p.m., 22:00 p.m., and 04:00 a.m., respectively) for further analysis. These tissues, including liver, epididymal white adipose tissue (Epi-WAT), perirenal white adipose tissue (Per-WAT), and mesenteric white adipose tissue (Mes-WAT) and gastrointestinal tract were collected at different time points (ZT2, ZT8, ZT14, and ZT20). The liver at four time points was used to detect the mRNA expression and protein expression. The WAT at four time points was used to compare the weight and only the Epi-WAT at ZT8 was subjected to hematoxylin and eosin (H&E) staining and scanning electron microscopy (SEM) analysis. Duodenum and jejunum at ZT8 and ZT20 were used to detect mRNA expression and histological analysis. The food intake was detected by the calculated the food consumption at ZT0 and ZT12 during constant seven days. All experimental procedures were performed and approved by the guidelines of the Ethics and Animal Welfare Committee of Beijing Normal University (Approval No. CLS-EAW-2015-002).

### 2.3. Cell Culture and Steatotic Hepatocyte Model Construction

We obtained L-02 cells from the Cell Resource Center, Peking Union Medical College (Beijing, China), and culture media was DMEM supplemented with 10% FBS and 1% penicillin-streptomycin at 37 °C with 5% CO_2_. The steatotic hepatocyte model establishment was performed as previously described [47,48]. Briefly, L-02 cells at 80% confluency were exposed to a 1 mM free fatty acid (FFA) mixture (OA:PA ratio, 2:1). Melatonin was prepared with dimethyl sulfoxide (DMSO) as stock solution and treatment concentration was 0.5 mM, which was added simultaneously with FFA in the cells (FM group). The control group was treated with vehicle. The treatment time was 48 h, and after then the cells were used for further analysis.

### 2.4. Histological Analysis

To make paraffin sections, Epi-WAT, liver, and duodenum were fixed in 4% paraformaldehyde, paraffin-embedde,d and sectioned at 5–7 μm. Hematoxylin and eosin (H&E) staining was performed according to standard methods. Liver frozen sections (8–10 μm) were stained with Oil Red O or BODIPY. Duodenum and jejunum frozen sections (8–10 μm) were stained with Hoechst 33342 and Nile Red. Steatotic L-02 cells were stained using BODIPY.

### 2.5. Scanning Electron Microscopy (SEM) Analysis

For the analysis, 200-mg Epi-WAT samples were fixed in 2.5% glutaraldehyde (SEM grade) for 1 h at room temperature and then for 24 h at 4 °C. After extensive rinsing in PBS, the samples were fixed in 2% gallotannic acid for 1 h, followed by 1 h in 1% osmium tetroxide alone. They were then dehydrated in alcohol and isoamyl acetate and sputter-coated in gold. Micrographs were obtained at a working distance of 14 mm and an accelerating voltage of 10 kV on a JEOL it300 microscope.

### 2.6. Biochemical Analysis

ELISA kits were used to evaluate melatonin (IBL international Gmbh, Hamburg, Germany), insulin (ALPCO, Salem, NH, USA) and leptin (BioKit, Inc., Beijing, China) in serum. Triglyceride (TG) and total cholesterol (TC) in serum, tissue, cells, or feces were measured using commercially available kits from Applygen Technologies Inc. (Beijing, China).

### 2.7. Glucose and Insulin Tolerance Analyses

For the intraperitoneal glucose tolerance test (IPGTT) and insulin tolerance test (ITT), mice were fasted overnight and then given an intraperitoneal injection of D-glucose (1.5 g/kg) or insulin (0.75 UI/kg; Novo Nordisk). Blood samples were harvested from the tail vein at different time points, and the blood glucose was measured using a glucometer (Johnson & Johnson, Shanghai, China). After testing serum fasting blood glucose and insulin concentrations, we calculated the homeostasis model index of insulin resistance (HOMA-IR: insulin × glucose/22.5) and the quantitative insulin sensitivity check index (QUICKI: 1/[log (insulin) + log (glucose)]), as previously described [49].

### 2.8. Western Blot Analysis

Equal portions of tissue protein lysates (10 μg) were subjected to 10% SDS-polyacrylamide gel electrophoresis (SDS-PAGE) and electroblotted onto Immobilon^®^-P transfer membranes (Millipore, Billerica, MA, USA). Then, western blot analysis was performed using standard procedures. The following antibodies were used: antibodies against fatty acid synthase (FAS, sc-20140), sterol regulatory element binding protein 1 (SREBP1-1, sc-367), free fatty acid uptake transporter (CD36, sc-9154), and stearoyl-CoA desaturase 1 (SCD1, sc-515844) from Santa Cruz, an antibody against brain and muscle ARNT-like factor, (BMAL1, ab93806) from Abcam and an antibody against *β*-actin (A5441) from Sigma. The band intensity was quantified using the ImageJ software.

### 2.9. Gene Expression Analysis

Total RNA was extracted using an RNAprep Pure tissue kit or RNAprep Pure cell kit (Tiangen, Beijing, China). The total RNA (2 μg) was then reverse transcribed to cDNA using the FastKing gDNA Dispelling RT SuperMix (Tiangen, China). Quantitative real-time PCR was performed in triplicate using the SYBR Green qPCR SuperMix (Transgen Biotech, Beijing, China) on an ABI Q6 instrument (Applied Biosystems, Waltham, MA, USA) according to the manufacturer’s instructions. Gene expression was normalized against the murine or human glyceraldehyde-3-phosphate dehydrogenase (GAPDH) housekeeping gene. The primers used are listed in Supplementary Table S2.

### 2.10. Gut Microbiota Analysis

Fresh fecal samples were collected before the end of the raising phase for gut microbial analysis. Bacterial genomic DNA was isolated from stool samples stored at −80 °C using a QIAamp DNA stool Mini Kit from Qiagen (Hilden, Germany) according to the manufacturer’s instructions. The 16S rRNA gene comprising the V3 and V4 regions was amplified by PCR using composite-specific bacterial primers, which are listed in Supplementary Table S2. High-throughput pyrosequencing of the PCR products was performed on an Illumina MiSeq platform at Biomarker Technologies Co., Ltd. (Beijing, China).

### 2.11. Statistical Analysis

The results are presented as mean ± SEM. The statistical analysis was performed using SPSS, version 20 (IBM, Armonk, NY, USA). Differences between groups were statistically analyzed using one-way ANOVA by the Kruskal–Wallis test, repeated measure (RM) two-way ANOVA and ordinary two-way ANOVA by Tukey’s multiple comparisons test for factors time and treatment, and were considered statistically with a level of *p* < 0.05.

## 3. Results

### 3.1. Melatonin Shows an Antiobesity Effect in HFD-Fed Mice with Constant Light

**Figure 1:**
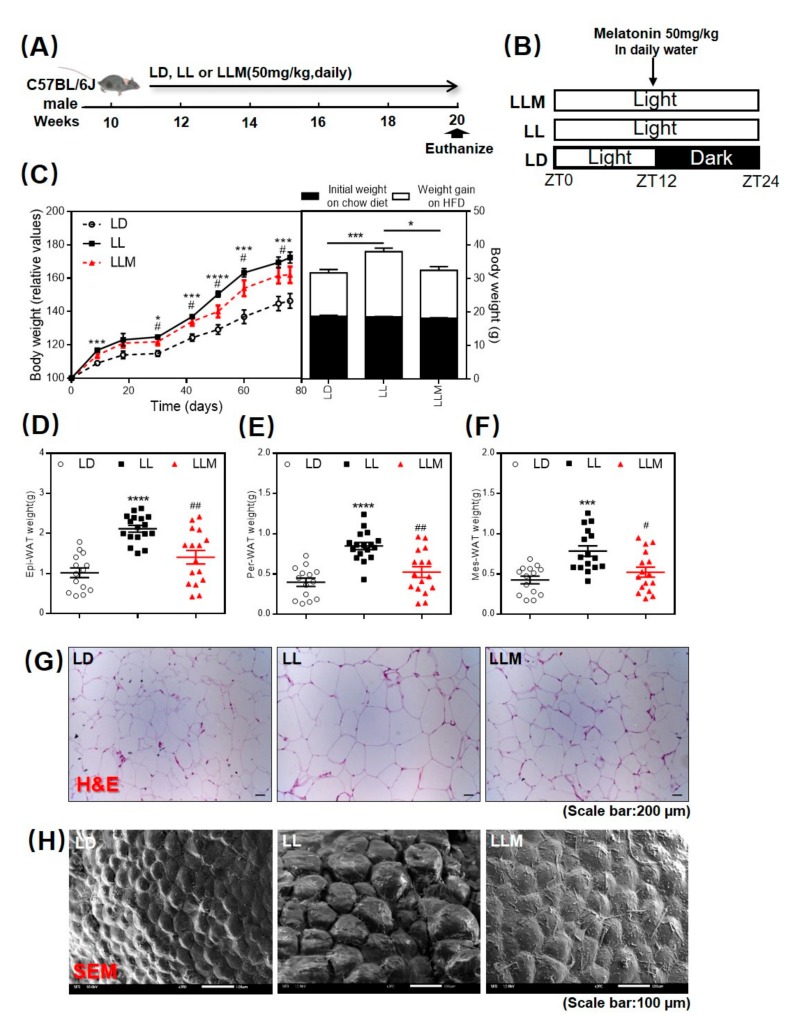
Melatonin shows an antiobesity effect in high-fat diet (HFD) fed mice with constant light. (**A**) Conceptual graph of the treatment group in mice. (**B**) Experimental design of the three groups. (**C**) Relative body weight value and body weight. (**D**) Epididymal white adipose tissue (Epi-WAT) weight, (**E**) Perirenal white adipose tissue (Per-WAT) weight, and (**F**) Mesenteric white adipose tissue (Mes-WAT) weight in the LD (12 h light-12 h dark cycle), LL (24 h light), and LLM (24 h light and treated with melatonin) groups. (**G**) Hematoxylin and eosin (H&E) staining of Epi-WAT sections (scale: 100 µm). (**H**) SEM of Epi-WAT sections (scale: 100 µm). Values are presented as the mean ± SEM. *N* = 16–20, * *p* < 0.05; ** *p* < 0.01; *** *p* < 0.001 compared with LD, ^#^ *p* < 0.05; ^##^ *p* < 0.01; ^###^ *p* < 0.001 compared with LL by one-way ANOVA.

To identify the effect of melatonin on body weight gain during constant light, 10-week-old male C57BL/6J mice were raised either with a 12-h light—12-h dark cycle (LD) or a 24-h light condition (LL) for 10 weeks. One-half of the mice in LL was supplemented with melatonin (LLM) simultaneously (50 mg/kg/d) in water but only during ZT12 to ZT24 every day (Figure 1A,B). Three groups of mice were fed an HFD for 10 weeks. As expected, the LL group mice gained more weight compared to the LD mice. Supplementation with melatonin showed a significant effect in preventing body weight gain under LL conditions (Figure 1C). We then isolated the three main types of adipose tissues from the mice: Epi-WAT, Per-WAT, and Mes-WAT. As shown in Figure 1D–H, consistent with the body weight gain, all three types of adipose tissue weight and adipocyte size detected by H&E staining (Figure 1G) and SEM (Figure 1H) were obviously increased in LL (*p* < 0.001) and decreased by melatonin supplementation in the mice.

### 3.2. Melatonin Decreases the Lipid Content and Ameliorates Insulin Sensitivity in HFD-Fed Mice with Constant Light

**Figure 2:**
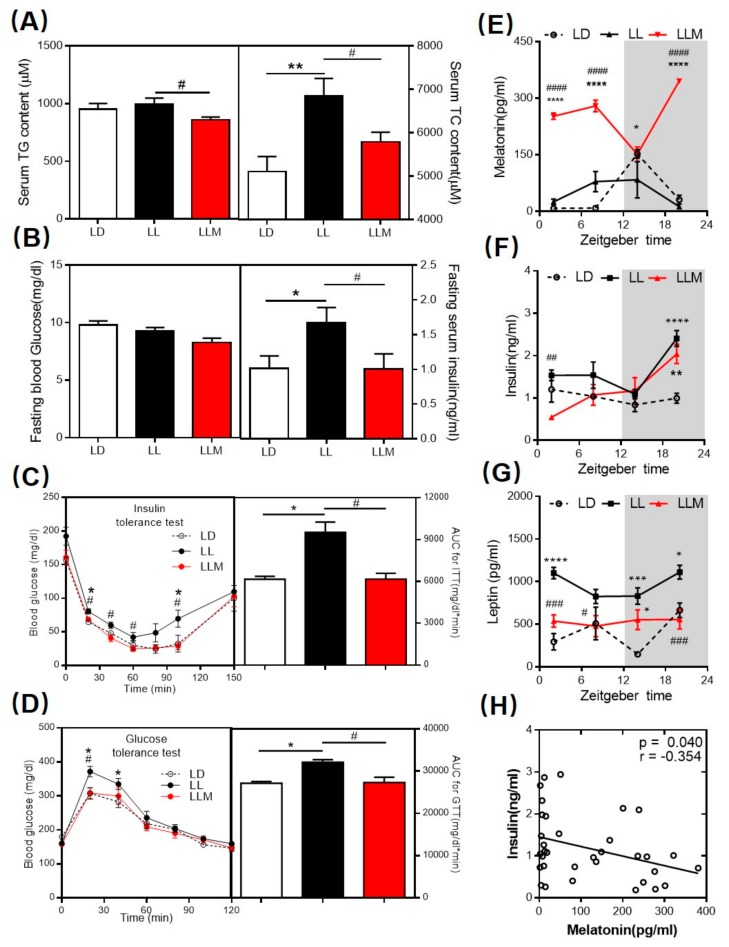
Supplementation of the melatonin level in the nocturnal phase can decrease serum lipid content and ameliorate insulin resistance in HFD-fed mice with constant light. (**A**) Serum triglyceride (TG) and total cholesterol (TC) content. (**B**) Fasting blood glucose and serum insulin levels. (**C**) Insulin tolerance test (ITT) and (**D**) glucose tolerance test (GTT) results. (**E**) Serum melatonin levels, (**F**) serum insulin levels and (**G**) serum leptin levels in mice after 24 h. (**H**) Correlations are depicted between the serum melatonin level and insulin level. Values are presented as the mean ± SEM. * *p* < 0.05; ** *p* < 0.01; *** *p* < 0.001 compared with LD, ^#^ *p* < 0.05; ^##^ *p* < 0.01; ^###^ *p* < 0.001 compared with LL by one-way ANOVA (**A**–**D**, *n* = 16–20) and repeated measure (RM) two-way ANOVA (**E**–**G**, *n* = 3–6).

To examine the ability of melatonin to attenuate hyperlipidemia, which was widespread in obese mice, we measured the serum lipid concentration. As shown in Figure 2A, after 10 weeks under different light conditions, the TC concentrations were significantly increased in LL compared to those in LD but were decreased by melatonin treatment. While the TG content was not changed by LL, melatonin treatment also mildly decreased the TG levels in serum. There is generally a strong association between obesity and insulin resistance. We measured the fasting blood glucose and insulin concentrations after 10 weeks of raising under different light conditions. Although all three groups of mice raised on HFD showed similar fasting blood glucose levels, LL had higher levels of fasting serum insulin, which is consistent with the results of the HOMA-IR and QUICKI (Figure 2B,F, and Supplementary Figure S1A). When challenged with additional insulin and glucose, melatonin administration clearly restored glucose with similar kinetics as those of LD mice, and the corresponding area under the curve (AUC) values further indicated that melatonin supplementation notably ameliorated the impaired glucose tolerance and insulin sensitivity of mice under constant light conditions (Figure 2C,D). Furthermore, we detected the melatonin and leptin levels in serum to confirm the endocrine hormone rhythm pattern under constant light conditions. As expected, melatonin was disturbed in LL, and melatonin supplementation reversed the level of serum melatonin in the nocturnal phase (Figure 2E). In obesity, serum leptin is usually associated with increasing adipose depots [50]. In our experiment, LL increased the leptin concentration at most ZT points, and melatonin treatment decreased the leptin level in serum (Figure 2G). Most interestingly, insulin and leptin concentrations were negatively correlated with melatonin levels in serum (Figure 2H and Supplementary Figure S1B).

### 3.3. Melatonin Decreases the Fat Accumulation in the Liver of HFD-Fed Mice with Constant Light

**Figure 3:**
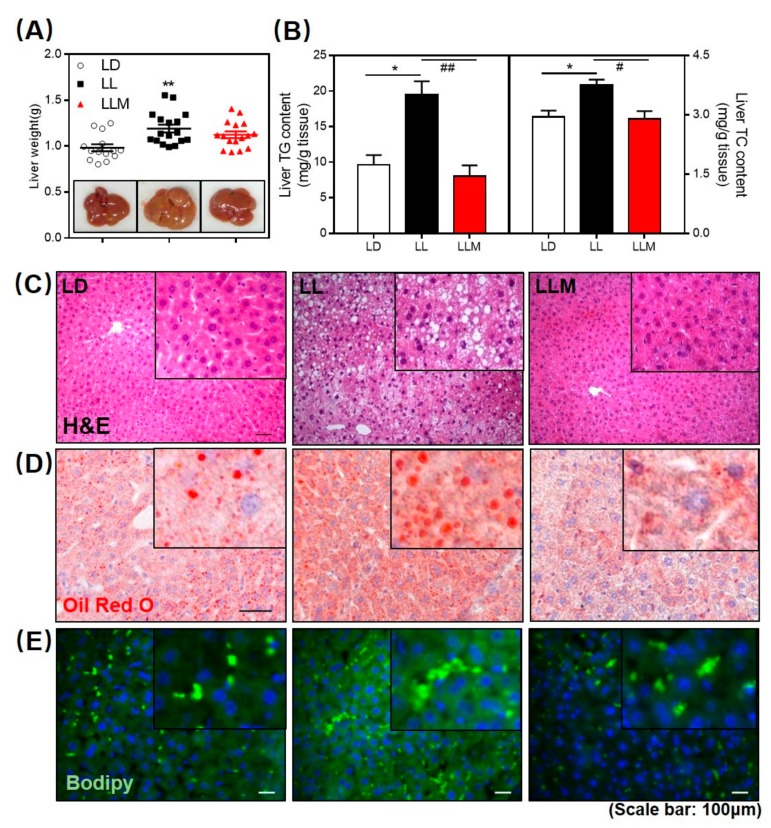
Melatonin decreases fat accumulation in the livers of HFD-fed mice with constant light. (**A**) Representative images of liver and liver weight. (**B**) Liver TG and TC contents. (**C**) H&E staining, (**D**) Oil Red O, and (**E**) BODIPY staining of liver sections (scale: 50 µm). Values are presented as the mean ± SEM. *N* = 16–20, * *p* < 0.05; ** *p* < 0.01; *** *p* < 0.001 compared with LD, ^#^ *p* < 0.05; ^##^ *p* < 0.01; ^###^ *p* < 0.001 compared with LL by one-way ANOVA.

Regardless of metabolic abnormalities, the obesity phenotype strongly increases the risk of developing nonalcoholic fatty liver disease (NAFLD) [51]. We found that liver weight was higher in LL, and the surface color of the liver was more yellow, as shown in Figure 3A. As expected, the TG and TC contents of the liver were significantly increased in LL mice, and melatonin treatment showed a beneficial effect in decreasing the lipid content in the liver (Figure 3B). H&E staining, Oil Red O staining, and BODIPY staining consistently indicated that melatonin effectively inhibited lipid infiltration in the mouse liver (Figure 3C–E).

### 3.4. Constant Light Changes the Rhythm Pattern of Clock Genes and Lipid Metabolism Transcripts

**Figure 4:**
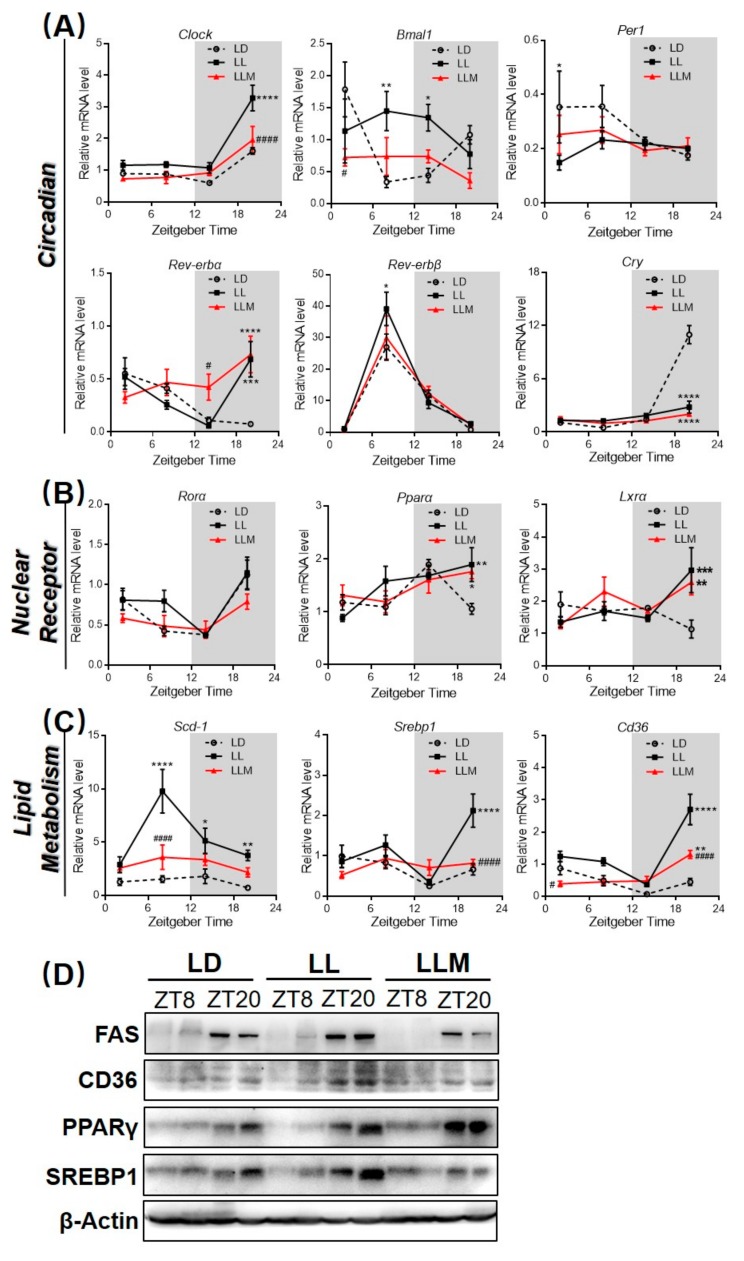
Constant light changes the rhythm patterns of clock genes and lipid metabolism transcripts. (**A**) The diurnal expression of various clock genes (*Clock*, *Bmal1*, *Per1*, *Rev-erbα*, *Rev-erbβ*, and *Cry*) was measured in the liver. (**B**) The diurnal expression of nuclear receptors (*Rorα*, *Pparα*, and *Lxrα*) was measured in the liver. (**C**) Transcripts involved in lipid metabolism (*Scd-1*, *Srebp1*, and *Cd36*) were measured in the liver. (**D**) The liver FAS, CD36, PPARγ, and SREBP1 protein production were examined by western blotting. Values are presented as the mean ± SEM. * *p* < 0.05; ** *p* < 0.01; *** *p* < 0.001 compared with LD, ^#^ *p* < 0.05; ^##^ *p* < 0.01; ^###^ *p* < 0.001 compared with LL by RM two-way ANOVA (**A**–**C**, *N* = 3–6).

To test the hypothesis that constant light affects clock genes expression, we analyzed the diurnal expression of transcripts encoding *Clock*, *Bmal1*, *Per1*, *Rev-erbα*, *Rev-erbβ*, and *Cry* in the liver after raising at different light conditions for 10 weeks (Figure 4A). *Bmal1*, *Rev-erbα*, and *Cry* mRNA expression showed markedly different diurnal rhythms in the LL compared with expression in the LD, while melatonin treatment attenuated this trend, which was caused by constant light. *Clock*, *Per1*, and *Rev-erbβ* mRNA showed synchronous expression patterns in the three groups, while LL significantly increased the expression of *Clock* at ZT20. In contrast to the circadian gene, LL had mildly changed mRNA expression of nuclear receptors, including *Rorα*, *Pparα*, and *Lxrα*, as shown in Figure 4B. Constant light increased the mRNA expression of *Pparα* and *Lxrα* only at ZT20. More interestingly, lipid metabolism-related genes were significantly changed under constant light conditions (Figure 4C). In particular, the fatty synthesis gene *Scd-1* diurnal rhythm was changed at ZT8 in which LL had a notably increased expression of *Scd-1* compared with that of LD, and melatonin treatment reversed this trend. *Srebp1* and *Cd36* also exhibited higher expression at ZT20 under constant light conditions, and this trend was inhibited in LLM, and further compared the protein expression of mouse liver among three groups. As shown in Figure 4D and Supplementary Figure S2C,D, compared with the LD, LL had increased FAS, CD36, and SREBP1 protein products at ZT20 in the liver. Additionally, melatonin treatment reversed lipogenic (FAS and SREBP1) and fatty transport (CD36) protein expression.

### 3.5. Melatonin Alleviates Lipid Infiltration in L-02 Cells by Reducing Lipogenesis Gene Expression

**Figure 5:**
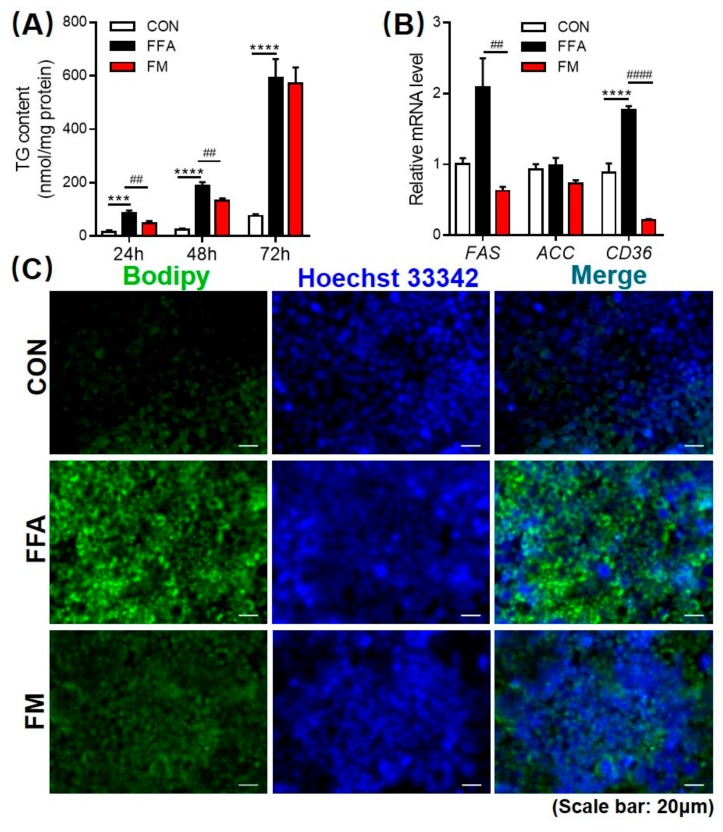
Melatonin alleviates lipid infiltration in L-02 cells by reducing lipogenesis gene expression. (**A**) Quantification of the intracellular triglyceride levels after melatonin treatment 24, 48, or 72 h. (**B**) The mRNA expression levels of fatty acid transport *CD36* and lipogenic genes *FAS* and *ACC* were measured by real-time PCR. (**C**) BODIPY staining of L-02 cells that were treated with a 1 mM FFA mixture (oleic acid and palmitic acid at a molar ratio of 2:1) for 48 h and also treatment with 1 mM melatonin (FM) (scale: 50 μm). Values are presented as the mean ± SEM. (**A**–**B**, *n* = 6) * *p* < 0.05; ** *p* < 0.01; *** *p* < 0.001 compared with LD, ^#^ *p* < 0.05; ^##^ *p* < 0.01; ^###^ *p* < 0.001 compared with LL by one-way ANOVA.

The L-02 cell model of FFA-induced steatosis was modified as previously described [49,52], and the TG content was measured after treatment with melatonin for 24, 48 or 72 h. The triglyceride content measurements and lipophilic dye BODIPY staining revealed that melatonin notably decreased intracellular lipid accumulation in a time-dependent manner (Figure 5A,C). In the hepatic cellular model of steatosis, the expression of fatty acid transport (*CD36)* and lipogenesis (*FAS*) genes was inhibited in L-02 cells after 48 h cultured in the FFA mixture and treated with melatonin (Figure 5B).

### 3.6. Melatonin Improves the Intestinal Morphology in Mice during Constant Light

**Figure 6:**
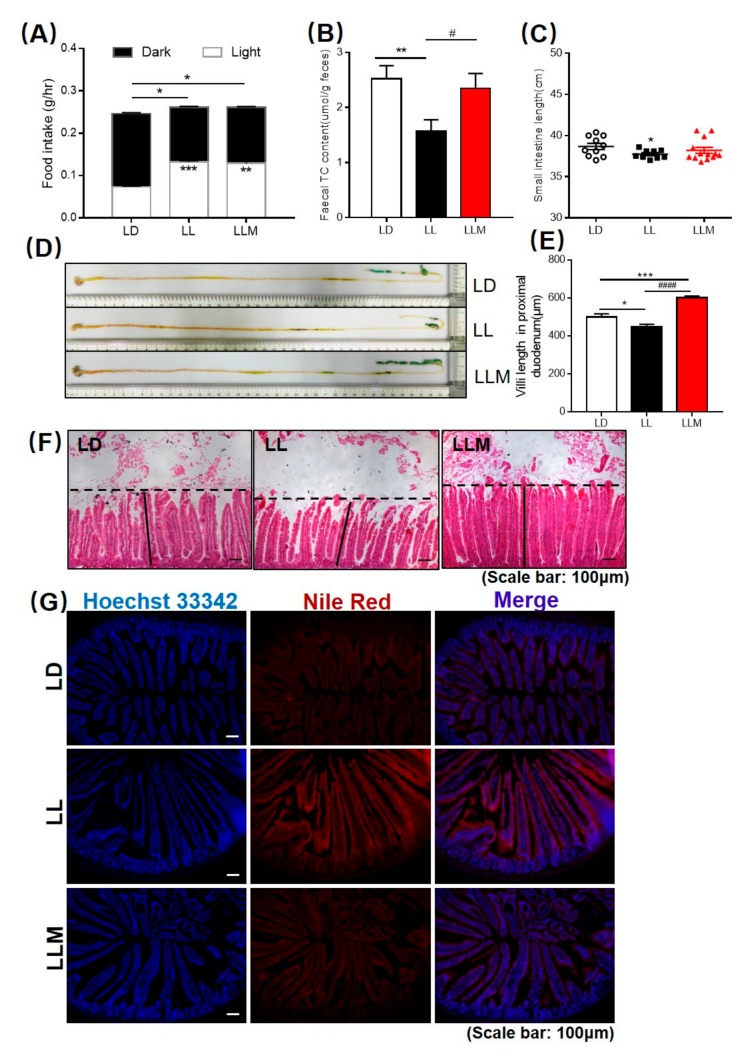
Constant lighting changes the pattern of food intake, and melatonin improves the intestinal morphology of mice. (**A**) Mice under the constant lighting condition ate more food during the diurnal phase than during the nocturnal phase. (**B**) Fecal TC content. (**C**) Small intestine length. (**D**) Representative images of the gastrointestinal system. (**E**) Proximal duodenum villus length. (**F**) H&E staining of duodenum sections (scale: 100 µm) (**G**) Nile Red staining of jejunum sections (scale: 100 µm). Values are presented as the mean ± SEM. N = 16–20, * *p* < 0.05; ** *p* < 0.01; *** *p* < 0.001 compared with LD, ^#^ *p* < 0.05; ^##^ *p* < 0.01; ^###^ *p* < 0.001 compared with LL by one-way ANOVA.

To investigate food consumption during different light conditions, we found that mice exposed to LL conditions ate mildly more food in total. In addition, the rhythm of food intake was obviously changed in the LL condition in which the mice ate more food during the diurnal phase than during the nocturnal phase. Such behavior is atypical in nocturnal animals such as mice (Figure 6A). We further measured the lipid content that is excreted in feces and stained lipid in the jejunum and found that melatonin treatment increased the TC content in feces and decreased the lipid present in the jejunum compared with the LL condition (Figure 6B,G). In addition, lipids were also increased in the duodenum under constant light conditions via Nile Red staining (Supplementary Figure S3D). To investigate changes in intestinal morphology, we measured villus length compared with LD controls. LL mice exhibited a markedly decreased small intestine length at sacrifice and shorter villi of the duodenum, whereas there were no differences in the weights of the whole gastrointestinal tract (Figure 6C–F and Supplementary Figure S3A,B). However, the length of the whole gastrointestinal tract in LL mice was also decreased (Supplementary Figure S3C).

### 3.7. Melatonin Improves the Gut Microbiota Composition in HFD-Fed Mice during Constant Light

**Figure 7:**
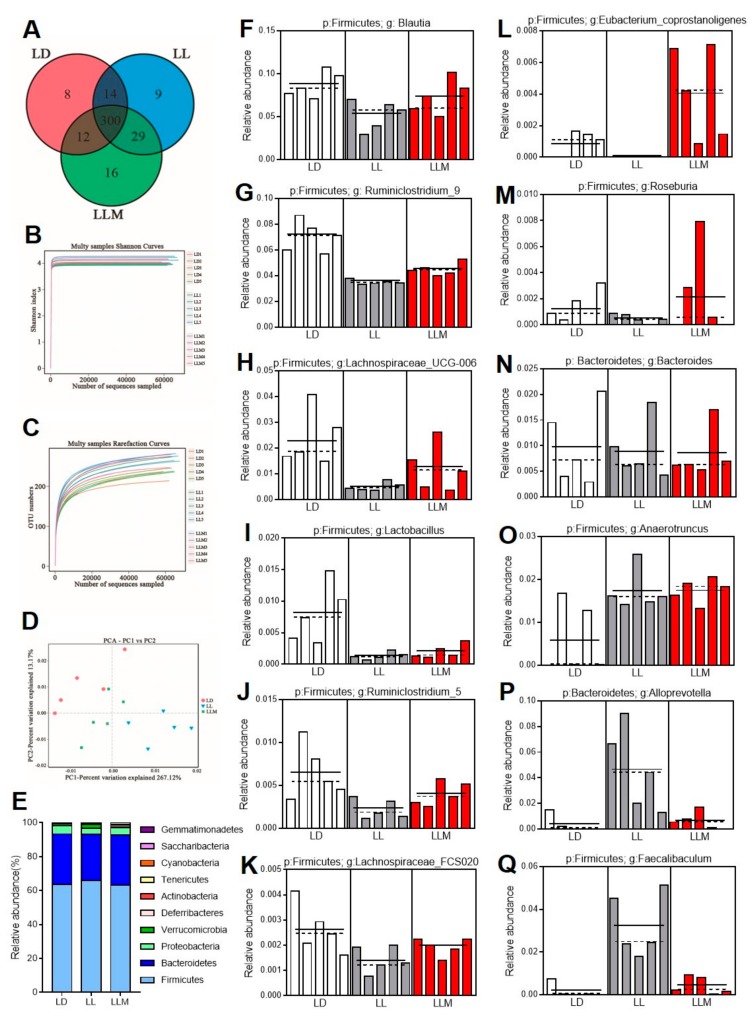
Melatonin improves the gut microbiota composition of HFD-fed mice with constant lighting. (**A**) Optical transform unit-Venn diagram (OTU-Venn), (**B**) Rarefaction curves, (**C**) Shannon curves, and (**D**) principal component analysis (PCA) of gut microbiota in the three groups. (**E**) Relative abundances of the gut microbiota at the phylum level. (**F**–**Q**) The relative abundance of *Blautia*, *Ruminiclostridium_9*, *Lachnospiraceae_UCG-006*, *Lactobacillus*, *Ruminiclostridium_5*, *Lachnospiraceae_FCS020*, *Eubacterium_coprostanoligenes*, *Roseburia*, *Bacteroides*, *Anaerotruncus*, *Alloprevotella*, and *Faecalibaculum* from the LEfSe results. *N* = 5, dotted line denotes median and solid line denotes mean.

Circadian rhythm disruption is one of the reasons for intestinal dysbiosis and is associated with various metabolic diseases, including obesity, diabetes and nonalcoholic fatty liver disease (NAFLD) [5,23,27,53]. In order to confirm the features of the microbiota that were influenced by constant light conditions, we performed a high-throughput pyrosequencing analysis of 16S rRNA in three groups. Based on 97% identity as the cutoff, 388 optical transform units (OTUs) were described, and the OTU-Venn diagram is presented in Figure 7A. The α-diversity analysis, including the rarefaction analysis and Shannon curves, indicated that there were no significant differences in the richness and diversity among the three groups in this study (Figure 7B,C). The intestinal microbiota structural changes were then performed by β-diversity analysis, including nonmetric multidimensional scaling (NMDS), UniFrac distance-based principal coordinate analysis (PCoA), and principal component analysis (PCA). As shown in Figure 7D and Supplementary Figure S4A,B, all three groups presented a distinct clustering of microbiota composition, and the LLM group had a similar structure to that of the LD group. As shown in Figure 7E, the phylum level analysis demonstrated that the mouse microbiota composition exhibited mildly different communities in the LD and LL conditions. A previous study demonstrated that obese mice had a decreased relative abundance of *Bacteroidetes* and increased relative abundance of *Firmicutes* [49], which was also shown in LL mice. Treatment with melatonin restored these levels and reduced the ratio of *Firmicutes* to *Bacteroidetes*. The top 100 relative abundance OTUs at the genus level are shown in Supplementary Figure S4C. Among the 100 OTUs, 54 OTUs were decreased in LL compared with LD, and melatonin treatment reversed 41 OTUs in the direction of those in LD mice. In addition, 27 OTUs were increased in LL compared with those in LD and LLM. In addition, 18 OTUs were specifically increased in melatonin treatment (Supplementary Table S3). To determine the specific bacterial taxa that were different between LD and LL, LEfSe analysis was employed (Figure 7F–Q). Detailed analysis of the OTUs that changed between LD and LL indicated that the changes in *Blautia* [54,55], *Ruminiclostridium* [56,57], *Lachnospiraceae* [58], *Lactobacillus* [56,59], *Eubacterium* [60,61], *Roseburia* [62], and *Bacteroides* [63,64] were all reversed by melatonin, and they all negatively correlated with obesity in previous studies. Interestingly, melatonin supplementation decreased the abundance of *Anaerotruncus* [65], *Alloprevotella* [66], and *Faecalibaculum* [67], which were all prone to enrichment in obese mice, as previously described. Collectively, our data suggested that melatonin treatment exhibited a beneficial effect in ameliorating gut microbiota dysbiosis induced by constant light in our model.

### 3.8. Melatonin Regulates Lipid Absorption and Excretion by Modulating Circadian and Lipid Transporter Gene Expression

**Figure 8:**
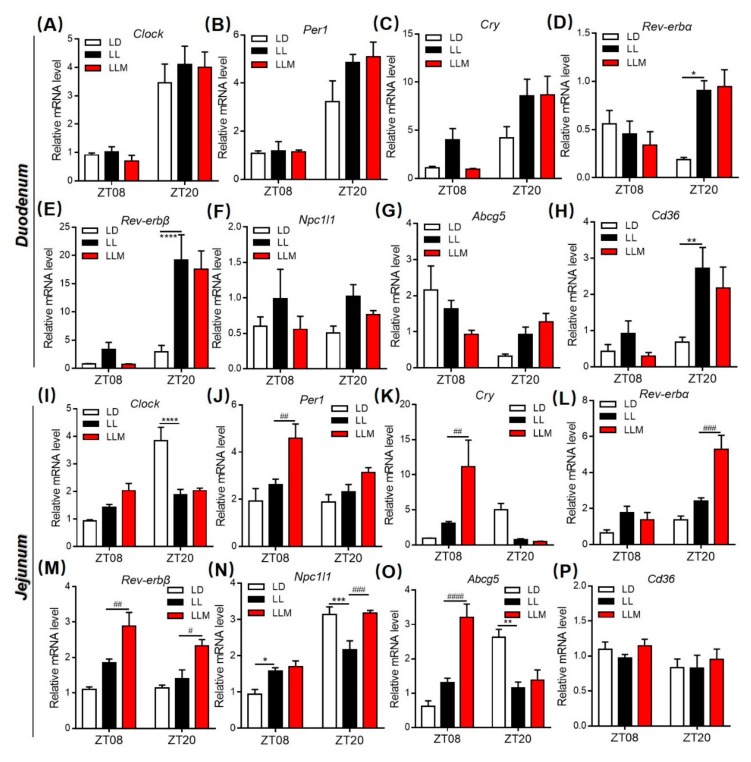
Melatonin regulates lipid absorption and excretion by modulating circadian and lipid transporter gene expression. (**A**–**H**) The expression of various circadian clock genes (*Clock*, *Per1*, *Cry*, *Rev*-*erbα*, and *Rev*-*erbβ*) and lipid transporter genes (*Npc1l1*, *Abcg5*, and *Cd36*) was measured in the duodenum at ZT08 and ZT20. (**I**–**P**) The expression of various circadian clock genes (*Clock*, *Per1*, *Cry*, *Rev*-*erbα*, and *Rev*-*erbβ*) and lipid transporter genes (*Npc1l1*, *Abcg5*, and *Cd36*) was measured in the jejunum at ZT08 and ZT20. Values are presented as the mean ± SEM. *N* = 3–6, * *p* < 0.05; ** *p* < 0.01; *** *p* < 0.001 compared with LD, ^#^ *p* < 0.05; ^##^ *p* < 0.01; ^###^ *p* < 0.001 compared with LL by two-way ANOVA.

To test the hypothesis that constant light affects *Clock* and lipid transport genes in the intestine, we analyzed the diurnal expression of transcripts encoding *Clock*, *Per1*, *Cry*, *Rev-erbα*, *Rev-erbβ*, *Npc1l1*, *Abcg5* and *Cd36* in the duodenum and jejunum at different light phase conditions for 10 weeks, as presented in Figure 8A–P. *Clock* and *Per1* expression showed no significant differences in the three groups in the duodenum. *Rev*-*erbα* mRNA expression showed a changed diurnal rhythm in the LL group compared with the LD group, while *Cry* and *Rev*-*erbβ* expression was increased in LL mice at two ZT in the duodenum. In the jejunum, *Cry* mRNA expression pattern in both LL and LLM groups was contrasted as that in LD. And melatonin treatment showed more effect in the jejunum, including increased *Per1*, *Cry* and *Rev*-*erbβ* mRNA expression at ZT08 and increased *Rev*-*erbα* and *Rev*-*erbβ* mRNA expression at ZT20. More interestingly, lipid metabolism-related genes were changed significantly under constant light conditions both in the duodenum and jejunum (Figure 8F,G,N–P). The cholesterol influx gene *Npc1l1* and the fatty acid transporter gene *Cd36* were increased in LL mice but reversed by melatonin treatment in the duodenum (ZT08 and ZT20). *Npc1l1* was increased in LL mice at ZT08 but decreased at ZT20, and melatonin treatment reversed the trend of that at ZT20 in the jejunum. Furthermore, the cholesterol efflux gene *Abcg5* showed the opposite expression pattern in the duodenum and jejunum. In the jejunum of the LL mice at ZT08, *Abcg5* expression was obviously different from that of the LD mice, and melatonin treatment strongly increased its expression.

## 4. Discussion

**Figure 9:**
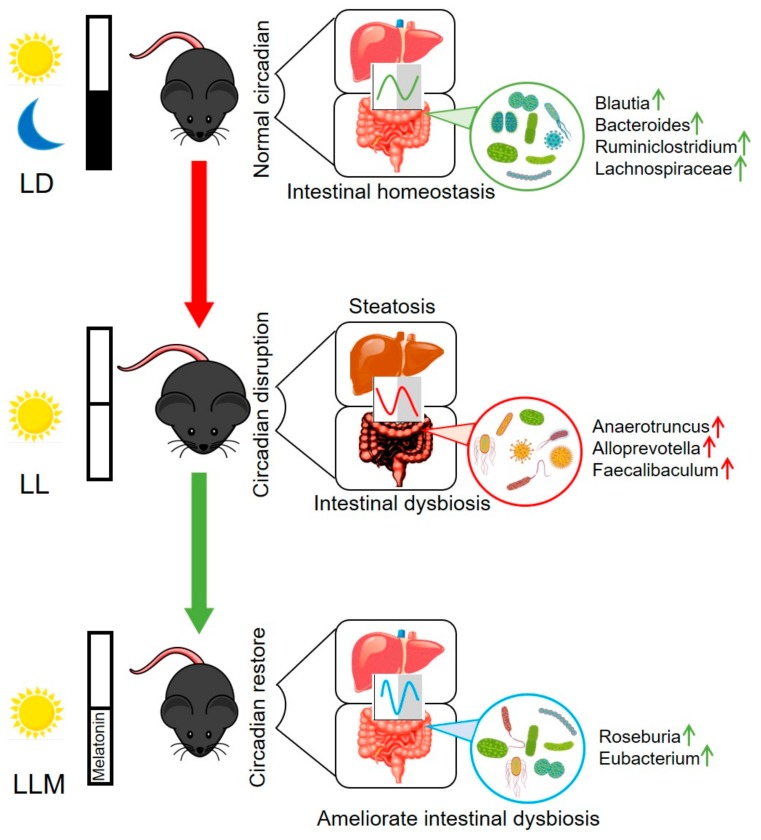
Schematic illustration of our proposed model. Melatonin restores the circadian clock and ameliorates intestinal dysbiosis during continuous light. LD: 12-h light–12-h dark cycle; LL: 24-h light condition; LLM: mice in the LL that were supplemented with melatonin daily (50 mg/kg/d) in water but only during ZT12 to ZT24. Mice were prone to obesity and showed metabolic disorders, including liver steatosis and intestinal dysbiosis, during LL conditions. Melatonin inhibited weight gain by modulating circadian rhythm and restoring microbiota composition in LL conditions. Red arrows represent bacteria that are prone to increase in obesity; green arrows represent prebiotic bacteria that are increased.

The hepatointestinal system plays the main role in nutrient absorption and metabolic waste excretion, and its function is closely related to obesity [20,68]. The healthy rhythm of the hepatointestinal system is vital for maintaining a balance of metabolic function [20]. Recently, increasing studies revealed that melatonin participates in maintaining homeostasis of the hepatointestinal system. For example, Sánchez DI et al. reported that melatonin prevented hepatocellular carcinoma by modulating dysregulated circadian clocks in mice [42]. Cassone VM’s laboratory demonstrated that GI-secreted melatonin regulates the circadian rhythm of commensal bacteria [46]. In addition, melatonin has shown beneficial effects in alleviating lipid dysmetabolism and weanling stress via modulating the composition of microbiota [37,38,45]. However, less is known about the regulatory effect of the circadian rhythm of the GI tract by melatonin. Long-term exposure to light at night was well documented, disturbing the circadian clock of the body and increasing the risk of obesity. In our model, exposure to 24 h of light, mice were prone to obesity and aggravated hepatic steatosis (Figure 9). LL mice exhibited tremendous changes in the rhythmical pattern of clock and lipogenesis genes in the liver after 10 weeks of exposure to constant light. Melatonin showed a beneficial effect in decreasing fat accumulation in the liver through inhibiting fatty acid synthesis and transportation. Additionally, 24 h of constant light disturbed the pattern of food intake and induced abnormal absorption and excretion of lipids in mice. The possible mechanism was that LL changed the expression pattern of circadian and lipid metabolism-related genes and exacerbated the dysbiosis of the gut microbiota, thus affecting lipid metabolism. Melatonin treatment efficiently prevented obesity induced by LL and inhibited lipid absorption but increased lipid excretion in the duodenum and jejunum by regulating GI circadian genes and restoring the composition of the microbiota.

Light as the most vital exogenous factor influenced the circadian clock and then modulated physiological activities such as food intake. Actually, constant light has already been applied in the poultry breeding industry to increase the feed intake amount and then accelerated the development of poultry, particularly in broiler breeding. Similar to this phenotype, people usually eat more food when they stay up and are consequently prone to obesity. Fonken et al. reported that constant light changed the pattern of food intake by increasing food consumption during the day phase in mice [8], which was also confirmed in our study. Under constant light conditions, mice had weight gain, an increase in three types of adipose tissue, as well as insulin resistance. Supplementation with melatonin alleviated the weight gain and improved the sensitivity of insulin in mice under the LL condition. As expected, mice also demonstrated a change in the pattern of food intake under long-term 24-h light exposure and showed almost the same food intake amount in the diurnal and nocturnal phases, which was different from the mice’s original nocturnal behavior. Increasing evidence has identified various adverse effects attributed to LAN. LAN-induced circadian misalignment exhibited a series of metabolic perturbations in peripheral tissues, including liver, intestine, and adipose tissue. To further investigate the correlation between circadian rhythm and nutrient absorption in GI, we detected the circadian genes (*Clock*, *Per1*, *Cry*, *Rev*-*erbα*, and *Rev*-*erbβ*) and lipid metabolism-related genes (*Npc1l1*, *Cd36*, and *Abcg5*) in the intestine. Constant light obviously changed circadian clock gene expression, especially in the jejunum. LL elevated *Clock* and *Rev*-*erbα*, while melatonin also increased *Per1* and *Rev*-*erbβ* at ZT08. At ZT20, LL decreased *Clock* and *Cry*, but melatonin increased *Per1*, *Rev*-*erbα*, and *Rev*-*erbβ*. LL inhibited the lipid excretion ability and then increased lipids present in the intestine, which was evidenced in the fecal TC content. Recently, Kuang Z et al. reported that microbiota influences diurnal rhythm and increased lipid uptake gene *Cd36* expression in the intestine by regulating histone deacetylase 3 (HDAC3) [69]. In our study, LL changed *Npc1l1* and *Cd36* expression, which are cholesterol and fatty acid transporters, respectively, in a diurnal phase in the GI tract. However, melatonin treatment significantly changed the rhythm of *Abcg5* expression, which regulates cholesterol excretion in the GI tract. The expression of *Abcg5* was tremendously increased in the diurnal phase in the jejunum, which could explain the effect of increasing lipid excretion by melatonin. More interestingly, the expression trend of *Abcg5* was positively related to *Cry*, which is a vital circadian gene. It was suggested that the regulation of *Abcg5* expression by melatonin could be involved in modulating *Cry* expression.

Increasing studies have demonstrated the relationship between microbiota and host homeostasis [70]. In our previous studies, dietary lipid adsorbent-montmorillonite (DLA-M) and the traditional Chinese medicine Danshensu Bingpian Zhi (DBZ) both showed antiobesity effects in high-fat diet-fed mice by improving the microbiota composition [49,71]. Recently, studies demonstrated that melatonin was also prebiotic that alleviated dyslipidemia and weanling stress [37,38,45]. Similarly, LL disturbed the circadian clock in mice and induced microbiota dysbiosis. As expected, melatonin reduced the ratio of *Firmicutes* to *Bacteroidetes* and restored the composition of the microbiota, including increasing the richness of *Blautia*, *Ruminiclostridium*, *Lachnospiraceae*, *Lactobacillus*, *Eubacterium*, *Roseburia*, and *Bacteroides*. Song et al. reported that *Blautia* was enriched in the effective weight loss group, but the relative abundance was different in different studies dependent on the species of this genus of bacteria [54,72]. In addition to butyrate-producing bacteria, *Roseburia* and *Lachnospiraceae* were decreased under constant light conditions in our model [62,73]. *Lactobacillus* is a well-documented probiotic strain for the human body that is widely used in the food industry and even as a probiotic therapy for children with acute infectious diarrhea [58,74]. It was reported that *Eubacterium* contributed to glucose utilization and intermediates of acetate and lactate fermentation [61]. Ridaura et al. demonstrated that *Bacteroides* could protect against obesity through fecal transplants from lean to obese mice [75]. Furthermore, melatonin treatment also inhibited the relative abundance of several genera of bacteria, such as *Anaerotruncus*, *Alloprevotella*, and *Faecalibaculum*. The relative abundance of *Anaerotruncus* was generally increased in HFD-fed mice, as previously reported [38,76]. Murugesan et al. reported that *Faecalibaculum* generally increased in overweight and obese children [67,77]. *Alloprevotella* has been shown to be negatively correlated with obesity and relative dyslipidemia [78]. However, there were still several bacteria that were disturbed under constant light conditions, such as *Akkermansia*, which is a well-known probiotic and was not restored by melatonin treatment in this study. The ability of lipid adsorption and efflux was changed by LL condition, and melatonin supplement showed beneficial effects in improving lipid efflux. The composition of nutrients especially dietary lipid could perform influence in metabolites in intestinal lumen where is an environment microbiota lived in. Thus, changed intestinal lipid metabolite by melatonin treatment could play a role in changing the abundance of microbiota as the potential molecular mechanism of beneficial effects. Additionally, improving the mucosal injury by melatonin also maintained the homeostasis of gastrointestinal and microbiota [30]. The protection effect of intestinal permeability by melatonin also found in our study (Figure 6D–F), then could as other potential reasons for the probiotic effect of melatonin. In all, the interference effect of constant light conditions or light at night and melatonin supplement on microbiota still needs further investigation.

The circadian rhythm of the liver is closely related to many types of disease [79]. Kettner et al. reported that chronic circadian disruption increased the morbidity of NAFLD, in particular making *Fxr*-/- mice prone to form non-alcoholic steatohepatitis (NASH) and even hepatocellular carcinoma (HCC), which revealed that disturbance of circadian rhythm was one of the causes of the formation of NAFLD [27]. Although all three groups of mice in our study were fed an HFD, the liver weight of the LL group mice was significantly higher than that of the LD group, and the lipid content of the liver was consistent with this phenotype. The liver tissue slices of the LL mice showed obvious fatty liver characteristics such as many lipid vacuoles in the liver through histological detection. Furthermore, we tested the gene expression pattern in the liver and found that circadian clock genes were changed tremendously in LL mice. In particular, *Bmal1* exhibited the opposite trend between LL and LD. In addition, lipid synthesis and transport genes, such *Scd*-1, *Cd36* and *Srebp1* were significantly increased in LL mouse livers compared with LD mice, which is consistent with the phenotype in LL mice. Melatonin has an effect on improving the pattern of circadian genes, especially inhibiting the lipogenesis genes *Scd*-*1* and the fatty acid transporter gene *Cd36* expression, thus alleviating fat accumulation in the liver. The effect of melatonin in inhibiting NAFLD has also been validated in a hepatic cell NAFLD model in which melatonin, decreased fat synthesis in L-02 cells. However, in order to confirm the positive effect of melatonin we chose a higher concentration in cells experiment, and it needs to further test to prove the effect of melatonin in human hepatocyte at the physiological concentration in the future study.

In conclusion, our study demonstrates that melatonin ameliorated lipid metabolic disorder induced by light rhythm disruption by regulating the circadian rhythm of the GI tract and the liver. Melatonin improved the pattern of circadian gene expression in the hepatointestinal system and improved the diversity and richness of the microbiota. In addition, melatonin inhibited the absorption of lipids and enhanced the excretion of lipids from the GI tract and alleviated fat accumulation in the liver.

## Supplementary Materials

The following are available online at https://www.mdpi.com/2073-4409/9/2/489/s1, Figure S1: Insulin sensitivity and correlations analysis, Figure S2: Lipid metabolism mRNA and protein level in liver, Figure S3: Gastrointestinal tract analysis and stain, Figure S4: Gut microbiota analysis, Table S1: HFD composition, Table S2: qPCR primer, Table S3: 100 top OUT, Table S4: Sample sequence result, Table S5: Phylum level OTU.
